# Regulation of TNF-Induced Osteoclast Differentiation

**DOI:** 10.3390/cells11010132

**Published:** 2021-12-31

**Authors:** Zhenqiang Yao, Stephen J. Getting, Ian C. Locke

**Affiliations:** 1 Department of Pathology and Laboratory Medicine, University of Rochester Medical Center, Rochester, NY 14642, USA; 2 School of Life Sciences, University of Westminster, 115 New Cavendish Street, London W1W 6UW, UK; s.getting@westminster.ac.uk (S.J.G.); I.C.Locke@westminster.ac.uk (I.C.L.)

**Keywords:** osteoclast, tumor necrosis factor alpha (TNFα), nuclear factor-kappa B (NF-κB), receptor activator of NF-κB ligand (RANKL), osteoprotegerin (OPG), TNF receptor-associated factor 3 (TRAF3), recombination signal–binding protein jκ (RBPjκ), interferon-regulatory factor 8 (IRF8), interleukin-1β (IL-1β), transforming growth factor-β1 (TGF-β1)

## Abstract

Increased osteoclast (OC) differentiation and activity is the critical event that results in bone loss and joint destruction in common pathological bone conditions, such as osteoporosis and rheumatoid arthritis (RA). RANKL and its decoy receptor, osteoprotegerin (OPG), control OC differentiation and activity. However, there is a specific concern of a rebound effect of denosumab discontinuation in treating osteoporosis. TNFα can induce OC differentiation that is independent of the RANKL/RANK system. In this review, we discuss the factors that negatively and positively regulate TNFα induction of OC formation, and the mechanisms involved to inform the design of new anti-resorptive agents for the treatment of bone conditions with enhanced OC formation. Similar to, and being independent of, RANKL, TNFα recruits TNF receptor-associated factors (TRAFs) to sequentially activate transcriptional factors NF-κB p50 and p52, followed by c-Fos, and then NFATc1 to induce OC differentiation. However, induction of OC formation by TNFα alone is very limited, since it also induces many inhibitory proteins, such as TRAF3, p100, IRF8, and RBP-j. TNFα induction of OC differentiation is, however, versatile, and Interleukin-1 or TGFβ1 can enhance TNFα-induced OC formation through a mechanism which is independent of RANKL, TRAF6, and/or NF-κB. However, TNFα polarized macrophages also produce anabolic factors, including insulin such as 6 peptide and Jagged1, to slow down bone loss in the pathological conditions. Thus, the development of novel approaches targeting TNFα signaling should focus on its downstream molecules that do not affect its anabolic effect.

## 1. Introduction

Bone is a dynamic structure that undergoes constant remodeling throughout life, in which mature bone tissue is removed by osteoclasts (OCs), a process called bone resorption, and new bone tissue is formed by osteoblasts (OBs), a process called bone formation. Imbalance of bone remodeling (increased OC bone resorption and/or decreased OB bone formation) results in bone loss, contributing to common diseases affecting adult bone, including osteoporosis [1,2,3], rheumatoid arthritis (RA), [4,5], bone metastatic cancer [6,7], aseptic loosening of arthroplasty [8], and periodontitis [9]. In postmenopausal osteoporosis, bone resorption is increased, associated with a coupled increase in bone formation [1,2,3], but bone formation relative to bone resorption is reduced [10], resulting in the accelerated bone turnover, and, finally, bone loss. Similarly, the ectopic differentiation and activation of OCs are essential for joint destruction seen in the inflamed joints of RA [4,5].

Several anti-resorptive and anabolic agents are available to treat osteoporosis. The widely prescribed anti-resorptive drugs, bisphosphonates [11], effectively increase bone mass, and reduce the rate of osteoporotic fracture by 50% [12]. As a result of the wider use of these therapies, the incidence of osteoporotic fracture has declined in developed countries [13]. For example, between 1980 and 2006, the overall incidence of hip fractures declined by 1.42% per year in women, and 0.44% per year in men in the Rochester area, US [14]. However, the rate of osteoporotic fracture is no longer declining, and for some fracture types, the rates have been rising in recent years [15].

Of concern is that bisphosphonates can result in atypical femoral fractures [16,17], hypocalcemia [18], and osteonecrosis of jaw bone [19,20] in a small number of patients. It is generally accepted that the incidence of jaw bone necrosis is rare in patients who take oral bisphosphonate, ranging from 0.01% to 0.99%. [21]. However, some reports have indicated that jaw bone necrosis is more frequent, approximately 4%, in osteoporotic patients taking long-term bisphosphonate orally [19]. The prevalence of bone necrosis is even higher in patients who are taking high-dose intravenous bisphosphonates [22]. Another anti-resorptive agent, denosumab (RANKL inhibitor), can also result in bone necrosis [23]. One special concern for denosumab is that its discontinuation is associated with a subsequent profound increase of bone turnover above pre-treatment values due to a substantial increase in OC number and activity, resulting in a risk of multiple vertebral fractures [24,25]. In addition, both bisphosphonates and denosumab do not effectively prevent focal joint destruction in human RA [26,27]. 

The anabolic agents include teriparatide (N-terminus 34 amino acid of parathyroid hormone) and newly FDA-approved abaloparatide (a parathyroid hormone-related protein analog) [28]. However, the effect of anabolic agents to increase bone mass is transient, and discontinuing these agents abruptly decreases bone mineral density (BMD) [29] due to increased bone resorption [30]. Therefore, the use of these anabolic treatments must be followed by anti-resorptive agents [28]. It has also shown that combined therapy with teriparatide and a bisphosphonate does not appear to offer advantages over the use of the single agent alone [31,32]. Similarly, switching from alendronate to teriparatide does not improve patients’ hip BMD, although the addition of an anabolic agent to ongoing alendronate does [33]. However, when patients established on potent bisphosphonates and denosumab are switched to teriparatide, the hip BMD declines below baseline for at least 12 and 24 months, respectively, after the switch to this anabolic agent [33]. Thus, for the osteoporosis treatment sequence, the anabolic therapy should be given first, followed by potent antiresorptive therapy. In addition, there are concerns that long-term use of anabolic agents may induce osteosarcoma, and their use is therefore generally limited to 1–2 years [34,35,36,37].

The newly approved romosozumab (a sclerostin monoclonal Ab) has a dual effect to increase bone formation early (and transiently), while inhibiting bone resorption persistently to treat osteoporosis [38]. However, it can increase the risk of severe side-effects, including myocardial infarction, stroke, and cardiovascular death [36]. Thus, romosozumab is only recommended for treatment of severe osteoporosis for up to 1 year [39].

In summary, the efficacy of current anti-resorptive and anabolic agents in treating osteoporosis and other bone diseases is limited, and their side effects have resulted in poor patient compliance, with many patients going untreated [40,41]. Thus, there is an unmet need to develop a new class of anti-resorptive drug for the treatment of diseases with enhanced bone resorption, including osteoporosis and RA. Considering the central role of OCs in bone diseases with bone loss, and the severe rebound effect of denosumab discontinuation, we review the signaling pathways for RANKL-independent OC differentiation in order to inspire the development of novel RANKL-independent anti-resorptive agents for the treatment of these bone diseases with bone loss.

## 2. Origin and Biological Characteristics of OC

An OC is a multinucleated cell with three or more nuclei, and is normally located on the trabecular surface. The cytoplasm of an OC has a high concentration of vesicles and vacuoles, including lysosomes filled with acid phosphatase (permitting the cell to be stained for the high activity of tartrate resistant acid phosphatase (TRAP)) and cathepsin K. An activated OC is characterized by cytoskeletal reorganization, the formation of sealing zones, and a specialized cell membrane, called a ruffled border, which opposes the surface of the bone tissue, allowing the secretion of acids and lysosomal enzymes onto the surface of bone to be resorbed [42,43]. Being deficient in sealing zone and ruffled border, as a result of defect of src kinase [44] or αv/β3 integrin [45], for example, incur OCs losing their resorptive function in mice. However, mutation of src kinase has not been identified in human patients with osteopetrosis [46,47]. Similarly, there is no report showing that an αv/β3 integrin genetic mutation causes abnormal OC function and osteopetrosis in humans, although defects in αIIb/β3 integrin causes Glanzmann thrombasthenia with bleeding in humans [48].

In postmenopausal osteoporosis, OC formation is increased with increased circulating bone resorptive markers [1,2,3], and OC resorptive function is also enhanced [49]. Normally, an OC does not present in the joint space, articular cartilage, or synovial tissue; however, in RA, OCs are ectopically differentiated and activated in the inflamed synovium and the adjacent articular cartilage where they actively erode cartilage and subchondral bone. OC formation is increased, and its function on the trabecular surface is enhanced, resulting in systemic osteoporosis in RA, particularly in patients treated with corticosteroids [50,51].

It has long been known that OCs originate from mononuclear phagocytes [52,53,54,55], which comprise monocytes/macrophages and dendritic cells (DC) originated from the common macrophage/DC progenitors (MDPs) [56]. Macrophage-colony stimulating factor (M-CSF) and its receptor, c-Fms, are required for macrophage generation [57], and for OC differentiation, since a deficiency of M-CSF results in osteopetrosis in mice, due to the lack of OC [58,59,60]. The mononuclear cells from any tissue, including bone marrow, spleen, thymus, or peripheral blood, can differentiate into TRAP^+^ OCs in vitro [61], and thus, monocytes/macrophages that have potential to form OCs are called OC precursors (OCPs). DCs can transdifferentiate toward functional OCs upon inflammatory conditions [62]. Thus, depletion of plasmacytoid dendritic cells inhibits osteolysis caused by breast cancer bone metastasis [63]. However, as a DC stimulating factor, granulocyte macrophage-colony stimulating factor (GM-CSF), strongly inhibits OC formation [64], although it stimulates the fusion of mononuclear OC precursors into bone-resorbing OCs by activating the Ras/ERK pathway [65].

OC differentiation from monocytes/macrophages requires M-CSF, but alone it is unable to complete the OC differentiation process [66]. In the late 1990s, two independent groups discovered that the receptor activator of nuclear factor-kappa B (NF-κB) ligand (RANKL), also named osteoprotegerin (OPG) ligand or osteoclast differentiation factor (ODF), is the additional factor that drives the terminal OC differentiation [67,68].

## 3. RANKL/OPG Signaling in Control of OC Differentiation

RANKL is a member of TNF superfamily of cytokine. Its receptor, RANK, with partial homology to a portion of the extracellular domain of human CD40, a member of TNF receptor superfamily, was discovered earlier to be involved in T-cell activation and dendritic-cell function [69]. Genetic deletion or mutation of RANKL or RANK results in severe osteopetrosis due to a complete OC defect [67,70]. When co-stimulated with M-CSF, the binding of RANKL with RANK in OCPs results in terminal OC differentiation, fusion, and activation. However, like other members of the TNF receptor super-family, RANK lacks intrinsic kinase activity to mediate downstream signaling [71]. The binding of RANKL to RANK recruits a variety of molecules, including the multifunctional adaptor proteins; TNF receptor-associated factors (TRAFs) 1, 2, 3, 5, and 6; and kinases, such as TGFβ-activated kinase-1 (TAK1), [71], to sequentially activate NF-κB p50 and p52, c-Fos, and NFATc1, the three sets of transcriptional factors that are required for OC differentiation [72]. NFATc1 is the master transcription factor regulating OC differentiation [73,74], and thus, over-expression of NFATc1 results in the terminal differentiation and fusion of OCPs without adding RANKL [73,74], and can rescue the defect of RANKL- and TNF-induced OC formation caused by the deletion of NF-κB p50/p52 or c-Fos [72,75].

OPG is a soluble decoy receptor of RANKL, and binds RANKL, blocking RANKL-RANK interaction, inhibiting OC formation and bone resorption [76]. Thus, genetic deletion of OPG leads to osteoporosis in mice [77,78], and homozygous deletion of 100 kilobases of *OPG* gene in human results in juvenile Paget’s disease, characterized by osteopenia and fractures [79].

RANKL/OPG ratio is thought to be a major determinant of OC differentiating status and bone mass [80]. It is thought that RANKL increases while OPG is decreased, leading to an increase in the ratio of RANKL to OPG, enhancing osteoclastogenesis in common bone diseases, such as osteoporosis and RA [80]. However, some published data indicates that serum levels of RANKL are reduced [81,82] while OPG is increased [55,83,84] with aging and in postmenopausal osteoporotic women. In addition, TNF-Tg mice generated to have a deficiency of RANKL still developed synovial inflammation, although joint destruction was blocked due to OC defect [4,5]. Clinical trials indicate that RANKL inhibitor, denosumab, did not alter RA disease activity [27,85], and did not suppress joint space narrowing, although it caused sustained suppression of bone turnover in RA patients [27]. These findings suggest that factors other than RANKL/OPG may play a role in enhancing OC differentiation in pathological conditions including osteoporosis and RA. It is important to elucidate the identity of the factors mediating enhanced OC formation, and to investigate their activation and mode of action resulting in the bone loss and joint destruction observed.

## 4. TNFα Induction of OC Formation Independent of RANKL Signaling

Kobayashi et al. [86] found that mouse bone marrow macrophages (M-BMMφ) formed TRAP^+^ OCs in response to TNFα in the presence of M-CSF, which was inhibited by antibodies against either TNF receptor 1 (TNFR1) or TNFR2, but not by OPG or anti–RANK antibody. Similarly, TNF-induced OC formation from M-BMMφ was impaired in the absence of either TNFR1 or TNFR2 [86]. In addition, a combined treatment of TNFα and TGF-β1 also induces OC formation from *RANk*^−/−^ OCPs in vitro [87]. These findings suggest that TNF-induced OC differentiation is independent of RANKL/OPG system.

**Figure 1:**
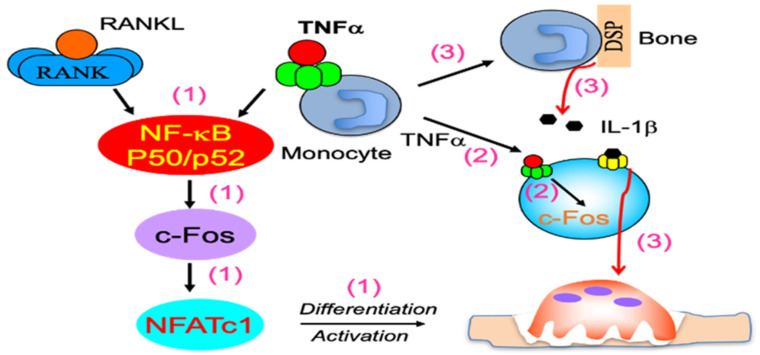
Key factors that mediate TNF-induced OC differentiation. (1) Similar to, and independent of, RANKL, TNFα sequentially activates NF-κB p50 and p52, then c-Fos, followed by NFATc1 to induce OC differentiation; (2) TNFα pre-activated OCPs expressing c-Fos attach to bone, and (3) are stimulated by bone matrix proteins, such as dentin sialoprotein (DSP), to produce IL-1β, which mediates the terminal differentiation of pre-activated OCPs into mature OCs to degrade bone.

Like RANKL, TNFα did not induce OC formation from primary spleen cells or M-CSF-induced macrophages (Sp-Mφ) of NF-κB p50/p52 double knockout (dKO) mice, suggesting that TNFα induction of OC differentiation requires the expression of NF-κB p50 and p52 [72]. Interestingly, and also similar to RANKL, TNF-induced c-Fos mRNA expression and activation was impaired in the dKO Sp-Mφ, and c-Fos over-expression rescued TNF-induced OC formation from dKO Sp-Mφ [72], suggesting that c-Fos is a downstream signaling of NF-κB for TNF- and RANKL- induced OC formation. Interestingly, over-expression of NFATc1, the down-stream transcriptional factor of c-Fos during OC differentiation [75], results in OC formation from both WT and NF-κB p50/p52 double knockout OCPs without the addition of RANKL or TNFα, and significantly enhances both RANKL and TNFα induction of OC formation from both WT and NF-κB p50/p52 double knockout OCPs [72]. These findings suggest that, like RANKL, TNFα sequentially activates transcriptional factors NF-κB, followed by c-Fos, and finally NFATc1, to induce OC formation. The potential of TNFα to induce OC formation is, however, weak [72], and TNFα pre-activated OCPs expressing c-Fos produce IL-1β in an autocrine mechanism by interacting with bone matrix protein, which further induces TNFα pre-activated OCPs to form mature OCs to resorb bone [88]. The key factors that mediate TNFα-induced OC differentiation are highlighted in Figure 1.

It was however puzzling that TNFα did not induce OC formation when it was administered in vivo to Rank knockout mice [89]. Lam et al. found that TNFα alone failed to induce OC formation from a pure M-BMMφ, whereas it dramatically stimulated their differentiation in the presence of permissive levels of RANKL [90], suggesting a synergistic effect of TNFα and RANKL on OC differentiation, and an important role of TNFα in OC formation in vivo on the bone surface where osteoblasts and osteocytes express permissive levels of RANKL [90]. Thus, it was proposed that TNFα induction of OC differentiation required the priming of OCPs by the permissive levels of RANKL in vivo, which explained why the administration of TNFα could not induce OC formation in vivo of RANK knockout mice. There is also, however, the possibility that TNFα induces unknown inhibitory factors to limit OC differentiation. Many negative and positive factors that regulate TNF-induced OC differentiation have in fact been identified, which are listed in Table 1 along with their reported functions.

## 5. Intracellular Factors That Limit TNFα Induction of OC Differentiation

### 5.1. Non-Canonical NF-κB2 Signaling Proteins

#### 5.1.1. NF-κB2 p100

**Figure 2:**
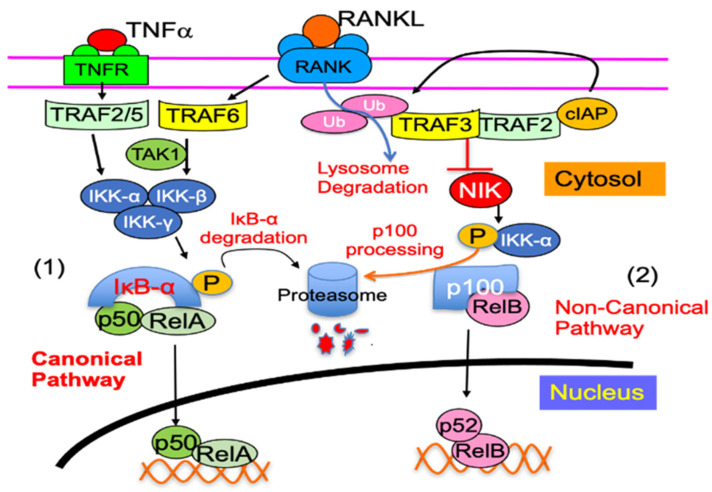
NF-κB signaling pathway. (1) Canonical NF-κB activation. Cytokines, such as RANKL and TNFα, induce canonical signaling by recruiting TRAF6 and TRAF2/5, respectively, to their receptors to activate a complex consisting of IKK-α, IKK-β, and IKK-γ (NEMO), which induces phosphorylation and degradation of IκB-α, and the release of p65/p50 heterodimers, which translocate to the nucleus to activate the target gene expression. (2) Non-canonical NF-κB activation. In unstimulated cells, the TRAF3–TRA2 complex results in constitutive NIK degradation, and thus, p100 binds RelB to be remained trapped in cytoplasm. Cytokines, such as CD40L and RANKL, recruit cIAP to the TRAF2–TRA3 complex, resulting in TRAF3 ubiquitin degradation, and allowing newly synthesized NIK accumulation. NIK then phosphorylates IKK-α, which leads to proteasomal processing of p100 to p52, releasing RelB:p52 heterodimers for translocation to the nucleus. TNFα does not degrade TRAF3, and thus, NIK is degraded, leading to the accumulation of p100 in the cytoplasm of osteoclast precursors to limit their differentiation.

NF-κB proteins include NF-κB1 (p50 and its precursor p105), NF-κB2 (p52 and its precursor p100), RelA (p65), RelB, and c-Rel. NF-κB protein homo- and heterodimers activate the transcription of target genes, typically through canonical (RelA:p50) and non-canonical (RelB:p52) pathways [113,114]. In the unstimulated cells, NF-κB dimers are retained in the cytoplasm by the inhibitory NF-κB proteins, called IκB, through their multiple ankyrin repeats [115]. Upon stimulation, IκBα is phosphorylated and degraded by a trimeric IκB kinase (IKK) complex, consisting of two catalytic subunits (IKKα and IKKβ) and a regulatory subunit, IKKγ [116,117], followed by the release of p50 from p105. As a result, RelA:p50 dimers are translocated to the nucleus to activate the target genes, which is called the canonical NF-κB signaling pathway [116], and occurs very rapidly, observed within minutes of stimulation. The key to non-canonical NF-κB signaling is the processing of p100 to p52 through NF-κB-inducing kinase (NIK). In unstimulated cells, NIK undergoes constitutive ubiquitin degradation by the TRAF3–TRAF2 complex. Upon stimulation, TRAF3 is degraded through ubiquitination, resulting in the accumulation of newly synthesized NIK [118], which phosphorylates IKKα. As a result, p100 is processed to p52 to release RelB:p52 heterodimers [119,120], which translocate into the nucleus, activating target genes. The key molecules that regulate NF-κB activation are highlighted in Figure 2.

The role of NF-κB in OC differentiation was discovered in the late 1990s when NF-κB1^−/−^ mice were crossed with NF-κB2^−/−^ mice to generate NF-κB1/2 double knockout (dKO) mice that developed severe osteopetrosis due to an OC defect [121,122]. However, single knockout of either NF-κB1 p50 or NF-κB2 p52 had normal OC numbers and function in vivo in mice [123]. This raises the question as to the role of NF-κB1 p105/p50, NF-κB2 p100/p52 in OC differentiation in physiological and pathological conditions.

Unexpectedly, the number of OCs induced by TNFα from spleen cells of NF-κB2^−/−^ mice was similar to that induced by RANKL from those of WT or NF-κB2^−/−^ mice in vitro [91]. Consistent with this, TNFα also induced a greater c-Fos and NFATc1 expression in *NFKB2*^−/−^ cells than WT cells, their expression level in *NFKB2*^−/−^ cells being similar to that induced by RANKL when mature OCs were forming [91]. Administration of TNFα induced significantly more OCs and a higher level of serum TRAP5b, a specific marker of bone resorption released by OCs, in NF-κB2^−/−^ mice than in WT mice [91], suggesting that NF-κB2 inhibits TNF-induced OC differentiation. NF-κB2 includes the precursor protein, p100, and the active form protein, p52. To determine if the inhibitory effect of NF-κB2 on TNF-induced OC differentiation is attributed to p100 or p52 over-expression, M-CSF-induced macrophages from WT and NF-κB2^−/−^ mouse spleen cells were over-expressed with p100 or p52 by the retroviral vector, followed by treatment with TNFα or RANKL in the presence of M-CSF. The results indicated that over-expression of p100, but not p52, significantly inhibited TNFα- and RANKL-induced OC formation, confirming that p100 is responsible for TNFα inhibition of OC differentiation [91]. This is consistent with earlier findings that TNFα-induced p100 was not processed to p52, and thus, was accumulated in the cells [124]. 

Similar to its effects in WT cells, TNFα also induced accumulation of p100 in *RANK*^−/−^ and *RANKL*^−/−^ OCPs. Interestingly, spleen cells from *RANK*^−/−^/*NFKB2*^−/−^ and *RANKL*^−/−^/*NFKB2*^−/−^ mice formed significantly more OCs than cells from *RANK*^−/−^/*NFKB2*^+/−^ and *RANKL*^−/−^/*NFKB2*^+/−^ mice in response to TNFα [91]. Although the osteopetrotic phenotype of RANK^−/−^ or RANKL^−/−^ mice was not changed by NF-κB2 gene deletion, local administration of TNFα induced many OCs and resorption lacunae in the calvarial bones of *RANK*^−/−^/*NFKB2*^−/−^ and *RANKL*^−/−^/*NFKB2*^−/−^ mice [91]. In contrast, only occasional OCs were observed in the sections of TNF-injected *RANKL*^−/−^ or *RANK*^−/−^ mice [91], as reported previously [89]. These findings provided the first in vivo evidence showing that TNFα induces functional OC differentiation, which is independent of RANKL signaling, whereas its ability to induce OC formation is limited by its induction of p100. 

A question that must be answered is whether there is a pathological significance for TNF-induced OCs in the absence of p100, since p100 deficiency does not change the osteopetrotic phenotype of RANKL^−/−^ mice [91]. TNF-Tg mice were crossed with *NFkB2*^−/−^ mice to generate TNF-Tg/*NFKB2*^−/−^ mice. Interestingly, they developed joint deformity earlier, with more severe joint inflammation and erosion than their TNF-Tg littermates [91]. TNF-Tg/*NFKB2*^−/−^ mice also had reduced trabecular bone volume and cortical thickness, associated with increased OC numbers and surfaces on the trabeculae compared with TNF-Tg/*NFKB2^+/−^* mice, as evaluated by histomorphometry and 3-dimensional μCT imaging [91]. However, serum TNF concentration in TNF-Tg/*NFKB2*^−/−^ mice did not increase compared with TNF-Tg/*NFKB2*^+/−^ littermate mice. These findings suggest that *NFKB2* deficiency accelerates joint inflammation and bone erosion in a TNFα-stimulated mouse model of RA. However, it is still unknown if *NFKB2* deficiency occurs in humans to involve in the pathological process in RA patients.

#### 5.1.2. TRAF3

The hallmark of non-canonical NF-κB activation is p100 processing into p52 by NIK, and TRAF3 inducing NIK constitutive ubiquitin degradation to negatively regulate non-canonical NF-κB signaling [125,126]. Interestingly, protein levels of TRAF3 parallel the change of p100 during RANKL- and TNFα-induced OC formation, with TNFα inducing a high protein level of TRAF3, whereas RANKL degrades it [91]. Importantly, treatment of OCPs with TRAF3 siRNA prevents TNFα- induced NF-κB2 p100 accumulation and inhibition of osteoclastogenesis [91], whereas over-expression of TRAF3 inhibits RANKL-induced OC formation directly [92]. These findings confirmed that TRAF3 negatively regulates OC differentiation by preventing p100 processing. Consistent with this specific deletion of *TRAF3* in myeloid progenitor cells in mice, results in mild osteoporosis associated with increased OC formation [92], further confirming that TRAF3 maintains bone homeostasis by inhibiting OC formation. 

The ubiquitinated protein can be degraded in proteasomes or lysosomes. CD40 or BAFF-R engagement in B cells induces rapid, proteasome-dependent TRAF3 degradation [127]. However, RANKL-induced TRAF3 degradation was not blocked by the proteasome inhibitor, MG132 [91], but was inhibited by the autophagy/lysosome inhibitor, chloroquine (CQ) [92]. Consistent with these findings, RANKL significantly increased co-localization of TRAF3 with LAMP2 (a lysosome marker), which was reduced by CQ [92]. Importantly, CQ also inhibited RANKL-induced OC formation from WT mouse OCPs, but not from *TRAF3*^−/−^ OCPs. Thus, RANKL degrades TRAF3 through the lysosome to promote OC differentiation. Interestingly, TRAF3 protein levels are reduced in the bone from aged mice and humans [94], which directly links TRAF3 deficiency to age-associated osteoporosis. TRAF3 also inhibits OC formation indirectly by limiting NF-κB mediated RANKL production by MPCs [94].

In contrast to RANKL, which efficiently degrades TRAF3 to promote OC differentiation, TNFα increases protein levels of TRAF3 [91], even in OCPs from TRAF6^−/−^ mice [93], which are osteopetrotic because TRAF6 is required for RANKL-induced OC formation [128,129,130]. Interestingly, RANKL is still involved in OC formation in the absence of TRAF6, because it degrades TNFα induction of TRAF3 [93], explaining why OCs are still present in the bone of TRAF6^−/−^ mice even though they are osteopetrotic [93,128]. However, the mechanism by which TNFα prevents TRAF3 degradation, or which factors other than RANKL can degrade TRAF3 to promote OC differentiation and bone loss are still unknown. Nevertheless, prevention of TRAF3 degradation could be a novel strategy for the treatment of osteoporosis and RA.

#### 5.1.3. RelB

Generally, NF-κB2 p100 associates with RelB, maintaining cytoplasmic localization, but upon stimulation, p100 is processed to p52, resulting in the release of the p52:RelB heterodimer, which translocates to the nucleus to activate target genes. However, TNFα increases p100 mRNA expression, but does not process it to p52, resulting in the accumulation of p100 [91]. Similarly, TNFα increases mRNA expression of RelB, but does not degrade it [98]. This raises the question of what the role is of RelB in OC differentiation. A report has shown that myeloid progenitor cells from RelB^−/−^ mice have an impaired OC formation in response to RANKL in vitro, but it is not understood why RelB^−/−^ mice have normal OCs on their bone in vivo [131]. In fact, RelB acts as a dual factor for OC differentiation [98]. TNFα elevates RelB protein to promote the differentiation of inflammatory macrophages with enhanced OC forming potential [98], but elevated RelB also inhibits the terminal OC differentiation by inhibiting NFATc1 expression [98]. TNFα stimulation therefore increases the pool of OCPs, but alone it induces very limited OC formation. RANKL, however, can degrade the excessively produced RelB to stimulate OC differentiation [98].

### 5.2. Notch Signaling Proteins

Notch is a family of evolutionarily conserved receptors that regulate cell fate. There are four different Notch receptors, Notch 1 to 4, and five ligands, Jag1, Jag2, Delta-like 1, 3, and 4, in mammalian cells [132]. Upon ligand binding, the Notch receptor intracellular domain (NICD) is cleaved by γ-secretase, and translocates to the nucleus, where it associates with the recombination signal-binding protein jκ (RBPjκ), leading to transcriptional activation or inhibition of target genes, such as *Hes1* and *Hey1* [132,133]. 

Notch signaling can directly regulate OC differentiation. Notch ligands downregulate cFms expression on OCPs to inhibit their differentiation into OCs [99]. Thus, deletion of Notch 1–3 in macrophages promotes their differentiation to mature Ocs in response to a low dose of RANKL, and these macrophages undergo faster proliferation in response to M-CSF compared to WT control cells [134]. RANKL also induces macrophages to express Notch2, which is recruited to and interacts with RelA on NFATc1 promoter driving its expression [135]. Notch ligands also inhibit the expression of M-CSF and RANKL by stromal cells to negatively regulate OC differentiation in vitro [99]. Consistent with this, specific activation of canonical Notch in osteocytes increases bone mass in mice, associated with reduced OC formation, and bone resorption, associated with decreased RANKL expression in these cells [100]. In contrast, osteoblast-specific loss of function of Notch led to age-related bone loss in mice, due to increased bone resorption caused by reduced OPG expression [101]. These reports generally suggest that Notch inhibits OC differentiation directly from OCPs by downregulating their expression of cFms, and indirectly in osteoblast/osteocytes by reducing RANKL and/or increasing OPG production. 

**Figure 3:**
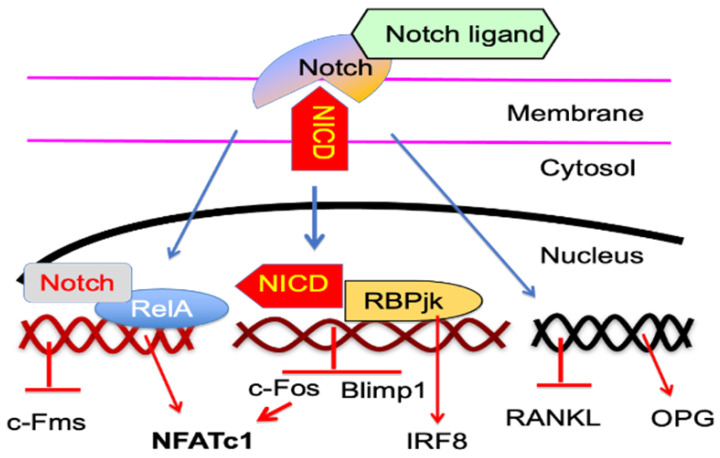
Notch signaling regulation of OC differentiation. Notch ligand binding to Notch receptors results in the cleavage and release of Notch NICD. As a result, NICD translocates into the nucleus, where it associates with RBPjκ and NF-κB to regulate the transcription of genes that control OC differentiation. NICD association with RBPjκ inhibits the expression of NFATc1 by attenuating c-Fos, and suppressing Blimp1, and promotes IRF8 expression. Notch also inhibits cFms expression, but associates with NF-κB RelA to promote NFATc1 expression. In addition, Notch inhibits OC differentiation, indirectly downregulating RANKL, while promoting OPG expression by MPCs and osteocytes.

TNFα mainly drives enhanced OC formation and bone resorption in pathological conditions, raising a question as to the role of Notch signaling in TNF-induced OC differentiation. Zhao and colleagues found that RBP-J, like p100, is a key negative regulator of osteoclastogenesis that restrains TNFα-induced excessive bone resorption in inflammatory settings [95]. In the absence of RBP-J, TNFα effectively induced osteoclastogenesis and bone resorption in RANK-deficient mice, and, in contrast, activation of RBP-J specific in OCPs suppressed osteoclastogenesis and arthritic bone resorption in TNF-Tg mice [95]. Further studies indicate that RBP-J suppresses the induction of NFATc1 by attenuating c-Fos activation, and suppressing the induction of B lymphocyte-induced maturation protein-1 (Blimp1), and thus, prevents the downregulation of transcriptional repressors, such as IRF-8, which blocks OC differentiation [95]. This report further confirmed the concept that TNFα-induced OC differentiation is independent of RANKL, but its ability to induce OC formation is limited by its induction of negative regulators [91]. However, similar to p100 knockout [91], the absence of RBP-J does not change the osteopetrotic phenotype of RANK^−/−^ mice, suggesting that RBP-J has minimal effects on physiological bone remodeling [95]. The mechanisms by which Notch signaling regulates OC differentiation are outlined in Figure 3.

## 6. Regulation of OC Forming Potential through Macrophage Polarization

Macrophages are classified as classically activated (inflammatory, M1) macrophages or alternatively activated (resident, M2) macrophages [57,136], which are linked to T helper 1 (Th1)- and Th2-type immune responses, respectively [137]. M1 macrophages mediate inflammatory responses to a variety of bacterial, protozoal, and viral infections, and produce many inflammatory cytokines, including TNFα, IL-12, IL-18, and IL-23, in several chronic inflammatory and autoimmune diseases, including RA, Crohn’s disease, multiple sclerosis, and autoimmune hepatitis [138,139]. M2 macrophages, in contrast, inhibit the production of a wide variety of pro-inflammatory mediators, through the production of cytokines, such as IL-10 and TGF-β, and thus, regulate wound healing [140]. Thus, it is widely accepted that targeted depletion of M1 and boosting activities of M2 macrophages are emerging as an attractive combined therapeutic strategy for autoimmune diseases [141,142,143]. However, a recent report shows that TNF-polarized macrophages (TPMs) produce insulin-like 6 peptide (INSL6) and Jagged1 to stimulate bone formation, slowing down bone loss caused by enhanced bone resorption in RA [144], which explains why anti-TNF therapy has a limited effect in improving the lost bone in patients with RA [145,146,147].

### 6.1. Macrophage Generation

Monocytes and macrophages, generated from myeloid progenitor cells in bone marrow, are phagocytes. The circulating phagocytes in the blood are known as monocytes, which become different types of macrophages when they enter tissues. GM-CSF and M-CSF are the two cytokines that generate macrophages. GM-CSF drives myeloid progenitor cells, differentiating into both granulocytes and macrophages, as well as dendritic cells (DC), and thus, it is often employed in studies of DC development and function [148,149]. However, GM-CSF is not critical for macrophage development, since mice lacking GM-CSF do not have notable defects in tissue macrophages [150]. In contrast, targeted ablation of M-CSF or its receptor, c-Fms, causes severe depletion of macrophages in many tissues associated with the failure of OC formation and osteopetrosis [57], indicating that M-CSF does play a major role in the generation of macrophages. Importantly, M-CSF generated macrophages can be polarized to the cells with either a stronger M2 anti-inflammatory [102,151,152,153] or M1 inflammatory cytokine profile [102], and thus, they are called M0 macrophages [153]. Macrophage polarization can have a profound effect on their OC forming potential.

### 6.2. Polarized M1 Macrophages with Altered OC Forming Potential

IFN-γ and *lipopolysaccharide* (LPS) are traditionally considered as factors which polarize M1s [102], with GM-CSF and *TNFα* being the latest additions to M1 category of stimuli [98,102,152]. The hallmarks of M1 activation are iNOS expression and high levels of IL-12 with low levels of IL-10 production [102]. A recent report shows that TNF-polarized macrophages do not produce iNOS, although they express the major M1 surface markers [144]. RANKL can stimulate the expression of iNOS, which acts as an autocrine negative feedback mechanism to restrain RANKL-mediated osteoclastogenesis [154]. Similarly, IL-12, like IL-18, was shown to potently inhibit OC formation partially via Fas- and FasL-mediated apoptosis [106,107]. 

M-CSF-induced macrophages are polarized to M1 via stimulating transcription factors signal transducer and activator of transcription 1 (STAT1) and interferon-regulatory factor (IRF) family members, including IRF1, IRF5, and IRF8 [155,156], which are implicated in the differentiation of Th1 cells by regulating transcription of interferon. These transcription factors also inhibit RANKL-induced OC formation. STAT1 deficiency dramatically prevented *IFN*-γ, IFN-β, and LPS inhibition of RANKL-induced OC formation [103,108], suggesting that STAT1 mediates *IFN*-γ, IFN-β, and LPS inhibition of RANKL-induced OC differentiation. 

Zhao and colleagues reported that OCPs from IRF8^−/−^ mice underwent increased osteoclastogenesis in response to both RANKL and TNFα [96]. Consistent with this, mice deficient in IRF8 showed severe osteoporosis due to increased OC formation, and enhanced bone destruction following LPS administration [96]. These findings suggest that the expression of IRF8, like p100 and RBP-j, in OCPs limits TNF-induced OC differentiation. In contrast, RANKL can downregulate IRF8 to initiate OC differentiation [96]. Consistent with these, mutation of the IRF8 gene promotes OC formation to increase the susceptibility to tooth root resorption in humans [97].

Both IFN-γ and GM-CSF strongly inhibit OC differentiation [103,157]. M1 macrophages polarized by LPS in cooperation with IFN-γ do not form TRAP^+^ OCs, but fuse to form multinucleated giant cells (MGCs) [158,159]. M1 macrophages polarized by LPS alone also fail to form OCs in response to RANKL [104]. However, LPS stimulates OC formation from RANKL-primed pre-OCs indirectly by stimulating TNFα and IL-1β production, although LPS directly inhibits RANKL-induced OC formation [105]. TNFα alone weakly induces OC differentiation that is independent of RANKL signaling [72]. Different from M1 macrophages polarized by IFN-γ, GM-CSF, or LPS, TNF-polarized M1 are versatile and have enhanced OC forming potential in response to RANKL, and TNFα does not inhibit RANKL induction of OC differentiation in its polarized cells [98], although TNFα inhibits RANKL-induced OC formation from M-CSF-induced macrophages [91]. 

### 6.3. Polarized M2 Macrophages with Reduced OC Forming Potential

M-CSF generated macrophages mainly have the M2 phenotype [102]. Like Th1 cytokine IFN-γ, Th2 cytokine IFN-β also inhibits RANKL-induced OC formation [160]. Th2-linked M2 macrophages are not a uniform population, and are often subdivided into M2a, M2b, and M2c categories [102]. The common denominator of all three M2 subpopulations in mice are high IL-10 production, and low production of IL-12, accompanied by the production of enzyme arginase-1, which depletes L-arginine, thereby suppressing T cell responses and depriving iNOS of its substrate [102,161]. M2a macrophages are induced by IL-4 and IL-13, and produce TGF-β and IL-1Ra alongside IL-10. M2b macrophages are induced by LPS and immune complexes, and produce IL-1, IL-6, and TNFα, in addition to IL-10. M2c macrophages are induced by IL-10 and TGF-β, and produce large amounts of IL-10 and TGF-β such that they are often referred to as anti-inflammatory macrophages, and are involved in tissue repair and remodeling [102,161]. However, TGFβ1 enhances TNFα-induced OC formation, which is independent of TRAF6 [87,93]. Particularly, elevated active TGFβ1 with aging induces TRAF3 lysosomal degradation in mesenchymal progenitor cells to inhibit bone formation directly through GSK-3β mediated β-catenin degradation, and to promote OC formation indirectly through NF-κB mediated RANKL production [94], forming the basis of age-related osteoporosis.

IL-4, produced by Th2 lymphocytes, is the traditional trigger cytokine for M2 macrophages [102,161]. It directly prevents OC differentiation from OCPs and actin ring formation through the induction of STAT6 [109,110], and suppression of RANK, NF-κB, MAPK, c-Fos, and NFATc1 expression, as well as calcium signaling [109,110,162]. In addition, IL-4 indirectly suppresses osteoclastogenesis by inhibiting RANKL, but enhancing OPG expression by stromal cells [109,110]. Mice over-expressing IL-4 do, however, develop an osteoporotic phenotype in vivo due to inhibition of bone formation [163]. Thus, the net effect of IL-4 on bone turnover in vivo represents an integrated outcome of its influence on various cell populations.

IL-13 and IL-4 are closely related cytokines. Like IL-4, IL-13 is also produced by Th2 lymphocytes, and induces M2 macrophage polarization [97,111]. Similar to IL-4, IL-13 inhibits OC differentiation directly by inducing STAT6 phosphorylation in the myeloid cells, and indirectly by stimulating OPG while suppressing RANKL production by stromal cells [109,110].

IL-10 is highly expressed by all three subtypes of M2 macrophages, and it serves to inhibit inflammation by suppressing TNFα and IL-1β production and function [97,111]. IL-10 is a key negative regulator of bone resorption [111,112,164,165,166], and inhibits OC formation directly by suppressing RANKL-induced NFATc1, c-Fos, and c-Jun expression [111,112], and indirectly by reducing RANKL while increasing OPG expression by stromal cells [164].

IL-1β and TGFβ can be produced by activated M2 macrophages [97,111] and also by many other cell types, including platelets [167] and T cells [168]. TGFβ, in particular, is a bone matrix protein, which is released through bone resorption, and activated by the acidic environment generated by activated OCs [169]. Both IL-1β and TGFβ cooperate with TNFα to induce OC differentiation, and enhance bone resorption independent of RANKL signaling [87,88], and are discussed separately below. 

### 6.4. OC Forming Potential from Unclassified Polarized Macrophages

There are multiple macrophage phenotypes that cannot be readily classified into any of the M1 or M2 groups, for example, tumor-associated macrophages (TAM) and IL-17- induced atypical M2-like macrophage subpopulations [170]. It was reported that TAMs from human breast cancer samples formed OCs in both RANKL-dependent and -independent mechanisms [171]. Although TNFα can directly induce OC formation from TAMs in Ewing’s sarcoma in the presence of M-CSF [172], it is unknown if TNFα- induced OCs from TAMs have any difference with those from normal macrophages, because TNFα induces RANKL-independent OC formation from normal macrophages [72,86,87,88,91,93]. It is also unknown if IL-17 polarized macrophages can alter their potential to form OCs.

## 7. Cytokines That Enhance TNFα Induction of OC Differentiation

### 7.1. IL-1β

IL-1β has long been known to stimulate bone resorption [173]. It is produced by a variety of cell types at sites of inflammation in and around bones, including monocytes/macrophages, and mature OC themselves [174,175]. Like TNFα, it promotes RANKL expression by marrow osteoblastic stromal cells to induce osteoclastogenesis indirectly [176]. Wei and colleagues also found that IL-1β mediates TNF-induced osteoclastogenesis by enhancing stromal cell expression of RANKL [177]. These findings generally suggest that, unlike TNFα, IL-1β alone does not directly induce OC formation from OCPs [86], but synergizes with TNFα to promote OC formation [88]. However, IL-1β can directly induce OC formation from TNFα pre-activated OCPs expressing c-Fos, and this process is independent of NF-κB p50 and p52 [88]. Particularly, the pre-activated OCPs interact with the SIBLING (small integrin-binding ligand, N-linked glycoprotein) family of bone matrix proteins, such as dentin sialoprotein (DSP) and osteopontin (OPN) [178,179], to produce IL-1β in an autocrine mechanism, driving themselves’ to differentiate into mature OCs to resorb bone [88]. It is unknown if IL-1β enhances TNFα-induced OC formation through reducing any inhibitory factor proteins, including TRAF3, p100, RBP-J, and IRF8.

### 7.2. TGFβ1

TGFβ1 was reported to repress RANKL while increasing OPG expression by osteoblasts to indirectly inhibit OC formation [180,181]. Since TGFβ is released from the bone matrix due to bone resorption, and is activated in the acid environment generated by the activated OCs, its reduction of RANKL and increase of OPG production thus acts as feedback signal to limit bone resorption [182]. In contrast, a low dose of TGFβ1 stimulates OC differentiation by increasing RANKL while reducing OPG expression by osteoblasts [180]. 

TGFβ1 was also reported to directly promote RANKL-induced OC formation [183,184], or even induce OC formation in the absence of RANKL [185]. It mediates the interaction between smad3 and TRAF6 in OCPs to promote RANKL-induced OC formation [186]. In addition, combined stimulation of TGFβ1 and TNFα induces WT mouse spleen cells to form a considerable number of TRAP^+^ OCs in the presence of M-CSF, and also induces RAW264.7 cells to form mature OCs [183]. As discussed above, TNFα alone induces OC differentiation from RANKL^−/−^ and RANK^−/−^ spleen cells [91]. Interestingly, TGFβ1 can enhance TNFα-induced OC formation from TRAF6^−/−^ spleen cells [93]. However, TRAF6^−/−^ mice are still osteopetrotic [87], suggesting that TGFβ/TNFα-induced OCs do not play major role in normal conditions. Nevertheless, TGFβ1 is one of the strong candidate factors that enhance TNFα-mediated OC formation and bone destruction in pathological conditions [72,91]. It is likely that TGFβ1 enhancement of TNFα-mediated OC formation is not through degrading TRAF3 [93], but it is unknown if TGFβ1 reduces any other TNFα- induced inhibitory factors, such as p100, RBP-J, and IRF8, in this process. 

## 8. Limitations of Anti-TNF Therapy to Improve Osteoporosis in RA

The risk of bone loss and fracture is increased in individuals with RA and other autoimmune diseases [187] because OCs, mediated by inflammatory factors, such as TNFα and IL-1β, induce not only erosion of cartilage and bone locally in affected joints, but also degradation of bone systemically [188]. Anti-TNF therapies have significantly improved the outcomes for RA patients in the past two decades, although they have not provided a complete cure or lasting remission [189,190]. Some reports show that TNF inhibitors increase BMD, associated with decreased markers of bone resorption and increased markers of bone formation [191,192]; however, other reports show that TNF inhibitors do not increase new bone formation [193]. Long-term treatment with TNF inhibitors even increases the rate of fracture, although BMD is increased in patients with ankylosing spondylitis [145]. Lee et al. found that anti-TNF therapy does not increase lumbar and femoral neck BMD in RA patients also receiving a bisphosphonate [146]. Lumbar and femoral neck BMD are even reduced after short-term anti-TNF therapy in patients with RA [147].

Many factors, including disease severity or activity and glucocorticoid treatment, which accelerates bone loss, could influence the effect of anti-TNF agents on bone mass in RA patients [146,187,194,195], and explain the discrepancies in these reported outcomes. In 6-week-old TNF-Tg mice with early-stage erosive arthritis, 4 weeks of TNF antibody treatment completely blocked the development of erosive arthritis, but only slightly increased vertebral bone mass, associated with a reduction in parameters of both bone resorption and formation [144]. Similarly, TNF antibody slightly increased trabecular bone mass in tibiae of 8-month-old TNF-Tg mice with advanced erosive arthritis [144]. Interestingly, TNFα increased osteoblast differentiation from bone marrow stromal cells containing a large number of macrophages, but not from pure mesenchymal progenitor cells (MPCs) [144]. Further analysis indicated that TNFα-polarized macrophages (TPMs) produced several anabolic factors, including Jagged1 and insulin-like 6 (INSL6). Consistent with this, anti-TNF Ab reduced INSL6 expression by macrophages in vitro, and in TNF-Tg mice in vivo. Importantly, inhibition of either Jagged1 or INSL6 blocked TNFα-induced osteoblast differentiation from bone marrow stromal cells [144]. This is consistent with the known function of Jagged1 that stimulates osteoblast differentiation, and maintains the osteoblastic osteoprogenitor pool [196,197]. Similarly, INSL6 functions to expand MPCs. Of note, TPM stimulation of osteoblast differentiation is unique, as this did not occur with resident and inflammatory macrophages polarized by IL-4 and interferon-λ [144]. These findings suggest that TPMs produce INSL6 and Jagged 1 to stimulate bone formation, and that anti-TNF Ab blocks not only enhanced bone resorption, but also the anabolic effect of TPMs on bone, limiting its ability to increase bone mass in this model of RA. 

## 9. Prospective

The RANKL/OPG system is critical to control OC formation [80], whereas TNFα appears to contribute pathological bone loss and joint destruction [198]. The potential of TNFα induction of OC differentiation is weak [72,91], since it also increases many inhibitory proteins, including TRAF3 [91,92], p100 [91], IRF8 [96], and RBP-j [95]. However, TNFα induction of OC differentiation is versatile. IL-1 or TGFβ enhances TNFα induction of OC formation [88,183], which is independent of the RANKL/RANK/TRAF6 axis or NF-κB [87,88]. Anti-TNF regents have significantly improved the treatment of RA; however, about 50% of RA patients do not respond to anti-TNF reagents [199,200]. It is not known if these patients have genetic variants of the inhibitory proteins, such as TRAF3, p100, and RBP-J, resulting in the loss of function of these proteins, thus limiting the role of anti-TNF reagents. In most cases, RANKL degrades these inhibitory proteins to induce constitutive OC differentiation [87,91,92,95], but it is not known if any other factor can decrease TNFα induction of these inhibitory proteins to enhance TNFα induction of OC formation and bone destruction. Nevertheless, elevation or stabilization of these inhibitory proteins, including TRAF3, p100, IRF8, and RBP-J, would be a novel strategy to treat a variety of bone diseases with bone loss, including osteoporosis and RA. It should be noticed that anti-TNF regents do not improve the lost bone in patients with rheumatoid diseases [145,146,147], because anti-TNF therapy not only blocks the enhanced bone resorption, but also the anabolic effects of TNF-polarized macrophages [144]. Therefore, targeting TNFα signaling to develop novel approaches in treating bone diseases with increased resorption should focus on its downstream molecules that do not block the anabolic effect from its polarized macrophages.

## Figures and Tables

**Table 1:** Factors that regulate TNF-induced OC differentiation.

Class	Factors	Functions
**Negative regulators**	TRAF3	Inhibits OC formation directly by preventing p100 processing [91,92,93], and indirectly by limiting RANKL production by MPCs [94].
	NF-κB2 p100	Inhibits OC formation [91] *.
	RBP-j	Suppresses NFATc1 to inhibit OC formation [95].
	*IRF8*	Inhibits OC formation [96,97].
**Positive regulators**	IL-1β	Promotes TNF-pre-activated OCPs expressing c-Fos to form OCs independent of NF-κB p50 and p52 [88].
	TGFβ1	Enhances TNF-induced OC formation independent of RANKL, RANK, and TRAF6 [87,93].
	*RANKL*	Degrades TRAF3 to enhance TNF-induced OC formation [93].
	NF-κB RelB	Mediates TNF-polarized inflammatory Mφ to enhance OC formation, but inhibits NFATc1 expression to limit terminal OC differentiation [98].
Untested factors known to regulate RANKL-induced OC formation **	Notch ligands	Inhibits OC formation directly by suppressing cFms expression by OCPs [99], and indirectly by reducing RANKL production [100].
	Notch intracellular domain	Limits OPG production by MPCs [101].
	*IFN*-γ	Polarizes M-CSF-induced resident to inflammatory Mφ [102], and strongly inhibits OC formation [103].
	*GM-CSF*	Polarizes inflammatory Mφ [102], induces DCs, and inhibits OC formation [64].
	LPS	Polarizes inflammatory Mφ [102], and inhibits OC formation [104], while promoting OC formation from pre-activated OCPs [105].
	IL-12	Inflammatory Mφ cytokine, inhibiting OC formation [106,107].
	IL-18	Inflammatory Mφ cytokine, inhibiting OC formation [106,107].
	STAT1	Transcription factor that polarizes inflammatory Mφ to mediate IFN-γ, IFN-β, and LPS inhibition of RANKL-induced OC formation [103,108].
	IL-4	Cytokine that polarizes resident Mφ through STAT6 to inhibit OC formation and activity [109,110].
	IL-13	Works similarly with IL-4 to inhibit OC formation [109,110].
	IL-10	Resident Mφ cytokine that inhibits OC formation by suppressing RANKL-induced NFATc1, c-Fos, and c-Jun expression [111,112].

* Intact NF-κB1 and NF-κB2 is required for both RANKL- and TNF-induced OC differentiation [72]. ** Whether these factors regulate TNF-induced OC differentiation have not been tested.

## Data Availability

Not applicable.
